# Knowledge, attitude, and practice among healthcare workers towards COVID-19 outbreak in Nigeria

**DOI:** 10.1016/j.heliyon.2020.e05557

**Published:** 2020-11-18

**Authors:** Francis Enenche Ejeh, Adamu Saleh Saidu, Samuel Owoicho, Nanven Abraham Maurice, Solomon Jauro, Laura Madukaji, Kenneth O. Okon

**Affiliations:** a Department of Veterinary Microbiology, Medicine, Faculty of Veterinary Medicine, University of Maiduguri, Nigeria; b Department of Veterinary Public Health & Preventive Medicine, Faculty of Veterinary Medicine, University of Maiduguri, Nigeria; c Nigerian Field Epidemiology and Laboratory Training Program, Nigeria; d Department of Diagnostic and Extension, Nigerian Veterinary Research Institute, Vom, Nigeria; e Department of TB/HIV, APIN Public Health, Nigeria; f Department of Medical Microbiology, Federal Medical Centre, Makurdi, Nigeria

**Keywords:** Clinical research, Epidemiology, Infectious disease, Microbiology, Public health, Health profession, Virology, COVID-19, KAPs, Nigeria, Pandemic, Risk-factors, SARS-CoV-2

## Abstract

**Background:**

Severe acute respiratory syndrome-coronavirus-2 (SARS-CoV-2) infection is a global pandemic. Healthcare workers' (HCWs) role in patient management is predisposing and can serve as means of hospitals and community transmission. This study evaluated HCWs' knowledge, attitude, and practice towards COVID-19 in Nigeria.

**Methods:**

we carried out a cross-sectional survey among HCWs during the COVID-19 outbreak in Nigeria from March to June 2020. The study assessed 346 HCWs for Knowledge, attitude, and practice by using an online (Google form) self-administered questionnaire, based on a convinience sampling technique Data were retrieved and analyzed using descriptive statistics. Chi-Square and one-way ANOVA were used to measure association and difference among demographic variables. The relationship between knowledge, attitude, and practice was measured using Spearman's rho correlation test.

**Results:**

the mean knowledge score of the HCWs was 7.1 on a scale of 0–8. The correct overall rate of the knowledge questionnaire was 88.75%. Knowledge was gained mainly from television (35.0%) and social media (35.0%). The mean attitude score on a scale of 0–6 was 5.31 ± 0.39. Most (92.5%) participants were confident that Nigerian medical scientists would win the war against COVID-19. The majority (92.2%) of the respondents thought that SARS-CoV-2 was not a biological weapon. About 1 out of 5 respondents held that faith healing or prayer is the only cure for COVID-19. A vast majority of the HCWs were taking precautionary measures such as avoiding crowded places (94.2%), washing of hands (96.0%), and the use of personal protective equipment (91.6%) against SARS-CoV-2 infection. Nevertheless, only 3 out of 5 HCWs used a face mask when leaving home. There was a significant (p = 0.046) positive correlation (0.584) between knowledge and attitude.

**Conclusion:**

our results showed that HCWs in Nigeria had excellent knowledge and possessed a positive attitude and good practice towards COVID-19. However; there were areas where poor knowledge, negative attitudes and unacceptable practices were observed. We recommend continuous public health education of HCWs on SARS-COV-2 infection control and prevention.

## 1 Introduction

Coronaviruses (CoV) are enveloped, single-stranded RNA viruses that are responsible for flu-like symptoms characterized by severe acute respiratory symptoms, high morbidity and mortality. These viruses are of zoonotic origin, highly contagious, and infective and effective reproductive numbers [1, 2]. The two major coronaviruses that had attracted public health attention globally were severe acute respiratory syndrome coronavirus (SARS-CoV) in 2003, and Middle East respiratory syndrome coronavirus (MERS-CoV) in 2009 [3]. These viruses have high mortality and infectivity and were restricted to Asia, the Middle East, and spread to a few countries via the movement of people [4].

Genomic sequence analyses revealed that SARS-CoV-2 and other SARS-CoV are 94.6% similar in amino acid sequence and 80% nucleotide sequence similarity [5]. However, SARS CoV-2 is more related to bat SARS-CoV (96.2% nucleotide similarity) than human SARS-CoV (79.0%) [6, 7]. Because of these similarities, the novel coronavirus was named SARS-CoV-2 [8]. The clinical symptoms of COVID-19 include fever, body pain, dry cough, tiredness, sore throat, difficulty breathing, chest pain or pressure, loss of speech or movement, and gastrointestinal syndrome [9, 10], with people presenting the asymptomatic form of the disease in Nigeria and other African countries [11].

The World Health Organization declared COVID-19 outbreak a public health emergency of international concern (PHEIC-Pandemic) on January 30, 2020. Currently, COVID-19 has spread to over 200 countries and territories, with over 7.5 million cases and 4,19,568 deaths globally [12, 13] as of June 2020. In Nigeria, the first reported COVID-19 case was in Lagos on February 27, 2020, while the number of cases and death had gradually increased. As of June 12, 2020, COVID-19 cases in Nigeria have reached 15181 and 399 deaths, including healthcare workers [14].

Globally, healthcare workers are at the forefront in the containment of COVID-19 outbreak, diagnosis, and management of infected patients. Unfortunately, healthcare workers had also been the source and means of nosocomial and community transmission [15]. The burden of the disease in both developed and developing countries had worsened the response and management strategies due to inadequate provision of personal protection equipment for healthcare workers, environmental contamination, overcrowding, and inadequate provision of proper isolation facilities [16]. Thus, to mitigate the increasing number of COVID-19 cases require the HCWs' adherence to the recommended measures taken to prevent transmission. These measures are affected mainly by knowledge, attitudes, and practices (KAPs) of the frontline workers [17].

In sub-Saharan Africa, the number of laboratories-confirmed cases is still relatively low compared to other continents. This picture might be due to low testing capacity and a lack of an active surveillance system. The few confirmed cases require an excellent isolation centre, waste management, and environmental decontamination, counselling from the mental health and psycho-social support experts, and public health education. To achieve an effective response and curtailing the transmission within hospital and community settings, it may require the public health awareness campaign through risk communication and community engagement activities within the target population of the HCWs and host communities. Based on our observation, using a questionnaire administered study approach, we assessed the KAPs of healthcare workers towards the COVID-19 outbreak in Nigeria and the KAPs analyses as the way forward in policymaking.

## 2 Materials and methods

### 2.1 Study design, sample size, and participants

We conducted a cross-sectional survey among healthcare workers within the six geo-political zones in Nigeria from April to May 2020 in major cities in Nigeria due to the national response to the novel coronavirus outbreak in the country.

In line with the principles of social distancing and curtail the spread of SARS-Cov-2, an online survey (Google form; https://forms.gle/uUQCHhxGD3yAQqTo6) was used to collect data. Google form allows questionnaire design, data collection, descriptive analysis of results, and download data via an excel spreadsheet for further analysis.

Epi. Info TM was used to calculate the sample size of 346 subjects to fulfil our research objectives at a 95% confidence level. Other criteria for sample calculation include the assumption of a 50% prevalence of proper knowledge and attitude, 5% bound-on error, and a 10% non-response rate. We used a convenience sampling method. The study population eligible for participation in this survey were HCWs, including Medical doctors, Veterinary doctors, Public Health officers, pharmacists, medical laboratory scientists and nurses, and others. Nigerians nationals of age 20 years or more were invited to participate. We identified 22 active HCWs WhatsApp groups in the six geo-political zones of Nigeria and contacts of individuals in the contact list of the authors. A total of 2500 HCWs in the twenty-two WhatsApp groups and contacts of the authors were approached to participate in the survey. Response acceptance was closed when the required sample size of 346 was attained.

### 2.2 Ethical permit and consent note

The research ethics committee of the Borno State Hospital Services, Ministry of Health, Maiduguri, Nigeria, approved our study protocol (MHSEC/03/2020/00022). A consent note was not required. The submission of the online answer to the questionnaire was considered as consent to take part in the study.

### 2.3 The questionnaire

A survey instrument was designed based on previous KAPs study on COVID-19 [18], course material regarding coronavirus disease outbreaks including the current COVID-19 pandemic by WHO [13], and guidelines issued by Africa Centres for Disease Control and Prevention (Africa CDC) [19]. The questionnaire consisted of four (4) parts:

Part one (1). Demographic characteristics of respondents. Demographic variables include gender, age category, religion, years of service in healthcare, the region of residence, and speciality.
Part two (2). Knowledge of respondents on COVID-19. Questions in this part included eight questions on sources of COVID-19 information, transmission, prevention and control, zoonotic nature, myths, and biocontainment.
The knowledge questions consisted of both dichotomous and Likert scales. Likert scales were converted to dichotomous scale (strongly agree and agree = agree and was assigned a score of 1; neutral, disagree and strongly disagree = disagree with a score of 0). For dichotomous questions (yes or no, true or false and I don,t know), a correct answer was scored 1 point while an incorrect answer was scored 0 points.
The total knowledge score range from zero to eight; the mean knowledge score of 0–4.99 was considered poor knowledge and a mean score of 5–8 was considered good knowledge of COVID-19.
Part three (3). The attitude of respondents towards COVID-19. This part consisted of 6 questions on the wrong impression, faith healing, and confidence as it affects most regions in Nigeria because of the religious nature of the people of Nigeria.
Six dichotomous questions measured attitude among HCWs towards COVID-19 outbreak in Nigeria. A correct answer corresponded to a positive attitude and was scored 1 point while A negative attitude corresponded to an incorrect answer and was scored 0 points. Attitude score range from 0-6. A mean score of 0–3.99 was considered poor or unacceptable attitude while a mean score of 4–6 was assigned a good, acceptable or positive attitude.
Part four (4). The practice of respondents towards COVID-19. The questions in this part seek to collect data on methods of prevention and control of COVID-19.
Five questions measured practices of HCWs towards COVID-19. An incorrect answer was scored zero points while a correct answer was scored 1 point. The practice score range from 0-5. A mean practice score of 0–2.99 was considered poor, and a mean practice score of 3–5 was regarded as good practice.

### 2.4 Validation and pilot study

We conducted a pilot study to assess the validity and reliability of the questionnaire before its use. Initially, five experts in the field of epidemiology and research from two universities in Nigeria evaluated the questionnaire to assess the degree to which items in the questionnaires are relevant and can correctly measure the knowledge, attitude, and practice of the healthcare workers in Nigeria on the subject of COVID-19. We reviewed the questionnaire and effected the correction suggested by the experts. Afterword, the surveys were then sent to 30 participants who filled the questionnaire. The data were used to assess internal reliability using Cronbach's alpha. The results showed internal reliability (with Cronbach's alpha = 0.65).

### 2.5 Data analyses

Data were entered in Microsoft Excel and later imported into SPSS V.16 for statistical analysis. Inferential statistics were applied depending upon the nature of data and variables. We used Student's t and ANOVA tests to determine the relationship between mean knowledge score and mean attitude score and socio-demographic variables. In the case of a significant ANOVA test, post hoc analysis (LSD) was performed for multiple comparisons between every two categories. Chi-square tests were applied to find a difference in attitude and practice (good vs poor) by demographic characteristics. The authors used Spearman's rho correlation test to find the relationship between knowledge, attitude, and practice. The statistical significance level was set at p < 0.05 (two-sided).

We developed the formula below to calculate the mean knowledge and attitude scores:

$$\text{MS}=\frac{NR\;\times NQ}{nRd}$$

where:

MS = Mean score (Knowledge or attitude)

NR = Number of response by a particular variable, e.g. male.

NQ = total number of questions

nRd = total number of respondents.

## 3 Results

### 3.1 Demographic characteristics of study participants

Three hundred and forty-six (346) healthcare workers in Nigeria participated in this survey. Table 1 shows the demographic characteristics of the study participants. More than two-thirds of the respondents were male 249 (72.0%), 214 (61.8%) belong to the age group 30–39 years, those that had worked for 6–10 years in healthcare services were 145 (41.9%), HCWs in the Northeast and North Central Nigeria was 113 (32.7%) and 104 (30.1%) respectively. Veterinary doctors were 120 (34.7%), while Medical doctors represent 67 (19.4%).

**Table 1:** Socio-demographic Characteristics of Healthcare Workers (n = 346) participated in the Study regarding Knowledge, Attitudes, and Practices.

Variables	Frequency (n)	Percentage (%)
**Sex**		
Male	249	72.0
Female	97	28.0
**Age categories**		
20–29	36	10.4
30–39	214	61.8
40–49	75	21.7
50–59	21	6.1
>59	0	0
**Marital Status**		
Married	242	69.9
Unmarried	104	30.1
**Experience (years)**		
0–5	88	25.4
6–10	145	41.9
11–15	58	16.8
16–20	16	4.6
21–25	16	4.6
26–30	9	2.6
>30	14	4.0
**Religion**		
Christianity	244	70.0
Islam	96	27.7
No religion	6	1.7
**Geographical zone**		
Northcentral	104	30.1
Northeast	113	32.7
Northwest	30	8.7
Southeast	27	7.8
South-south	37	10.7
Southwest	35	10.1
**Speciality**		
Medical doctor	67	19.4
Veterinary doctor	120	34.7
Public health officers	49	14.2
Nurses	22	6.4
Pharmacist	16	4.6
Medical laboratory scientist	20	5.8
Others	52	15.0

### 3.2 Knowledge of healthcare workers towards COVID-19

**Figure 1:**
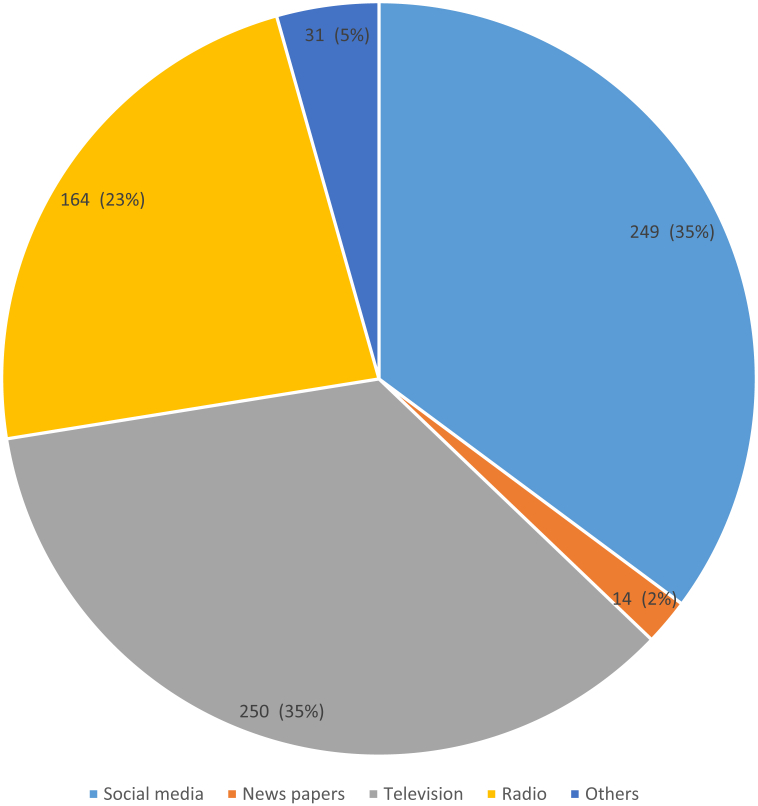
Sources of information on COVID-19 among Healthcare Workers in Nigeria.

All participants agreed to have heard about COVID-19. The most important sources of COVID-19 information were social media and television (Figure 1). The correct rate of the eight questions on knowledge range from 77.7 – 96.9% (Table 2). The overall knowledge score of healthcare workers in Nigeria was 7.1 (SD: 0.45; Range: 0–8), the correct overall rate of the knowledge score was 88.75% (7.1/8). Knowledge score significantly differs (0.019) among faithful of different religious bodies. Christianity and Islam had scores of 7.2 ± 0.46 and 7.0 ± 0.62, while HCW, who do not have religion scored 6.0 ± 1.23 (Table 3).

**Table 2:** Knowledge, Attitudes, and Practices of healthcare workers towards COVID-19 Outbreak in Nigeria.

S/No	Knowledge about COVID-19	Yes (%)	No (%)
1	Aware that COVID - 19 can affect both humans and animals?	269 (77.7)	77 (22.3)
2∗	Suspected persons should be isolated in a proper place for 14 days.	334 (96.5)	12 (3.5)
3	Isolation and treatment of confirmed cases of SARS-COV-2 can curtail the spread of the virus.	Agree 318 (91.9)	Disagree 28 (8.1)
4	believe that eating garlic or drinking much water can cure COVID -19?	56 (16.2)	290 (83.8)
5	Wearing of medical masks can help to prevent the infection by the COVID-19 virus.	316 (91.3)	30 (8.7)
6	Avoiding crowded places can prevent the spread of SARS-COV-2 infection.	311 (89.9)	35 (10.1)
7∗	There is no effective cure for COVID-19, but palliative treatments can help most patients recover from the disease	Agree 314 (90.8)	Disagree 32 (9.2)
8	SARS-COV-2 can be spread by Droplets by people, Airborne, Direct hand contact, Indirect hand contact, and Hugging	316 (91.3)	30 (8.7)
	**Overall Mean knowledge score = 7.1 ± 0.45**		
	**Attitudes towards COVID-19**	Positive (%)	Negative (%)
1	Africans are immune to COVID – 19 virus infection because of their genetic makeup	315 (91.0)	31 (9.0)
2	SARS-COV-2 was invented to reduce the human population in Africa.	319 (92.2)	27 (7.8)
3	Do you have confidence that medical scientists can win the battle against the COVID-19 virus?	320 (92.5)	26 (7.5)
4	Prayer (faith healing) is the only cure for COVID-19 infection.	271 (78.3)	75 (21.7)
5	Should people be allowed to congregate in a large crowd at a place of worship?	326 (94.2)	20 (5.8)
6	COVID – 19 is a punishment from God because people sinned against God	286 (82.7)	60 (17.3)
	**Overall Mean Attitude score = 4.35 ± 0.79**		
	Practices towards COVID-19	Good (%)	Poor (%)
1	Do you wash your hands after handling a sick person/animal?	340 (98.3)	6 (1.7)
2	Do you wash your hands after handling your pet or companion animal?	332 (96.0)	14 (4.0)
3	Do you wear protective clothing when handling a specimen from a person with sings of dry cough, sneezing, and fever?	317 (91.6)	29 (8.4)
4	How do you cough?	313 (90.5)	33 (9.5)
5	Do you wear a mask when leaving home?	205 (59.2)	141 (40.8)
	**Overall Mean Practice score = 5.31 ± 0.39**		

• Adapted from Zhong et al., 2020.

**Table 3:** Mean knowledge and attitude scores of healthcare workers towards COVID-19 in Nigeria.

Demographic Characteristics	Knowledge, N = 346			Attitude N = 346	
	Number (%)	Mean Score ±SD (0–8)	P Value	Mean Score ±SD (0–6)	P-Value
**Gender**					
Male	249 (72.0)	7.19 ± 0.48	0.429	5.28 ± 0.39	0.684
Female	97 (28.0)	6.99 ± 0.16		5.38 ± 0.44	
**Age group**					
20–29	36 (10.4)	6.81 ± 1.03	0.566	5.47 ± 0.37^a^∗	**0.014**
30–39	214 (61.8)	7.16 ± 0.44		5.43 ± 0.39^a^∗	
40–49	75 (21.7)	7.19 ± 0.47		5.09 ± 0.52	
50 and above	21 (6.1)	7.24 ± 0.58		4.57 ± 0.60^b^	
**Experience (year)**					
0–5	88 (25.4)	6.93 ± 0.75	0.094	5.05 ± 0.60^c^∗	**0.0001**
6–10	145 (41.9)	7.24 ± 0.37		5.61 ± 0.23^c^∗∗∗	
11–15	58 (16.8)	7.24 ± 0.22		5.31 ± 0.59^c^∗∗	
16–20	16 (4.6)	7.56 ± 0.82		5.13 ± 0.39^c^∗∗	
21–25	16 (4.6)	6.25 ± 1.17		5.50 ± 0.39^c^∗∗∗	
26–30	9 (2.6)	7.22 ± 1.11		5.56 ± 0.69^c^∗∗∗	
>31	14 (4.1)	7.29 ± 0.30		3.71 ± 1.04^d^	
**Health Profession**					
Medical doctor	67 (19.4)	7.22 ± 0.44	0.404	5.57 ± 0.34	**0.051**
Veterinary doctor	120 (34.7)	7.27 ± 0.37		5.48 ± 0.19	
+Public Health	49 (14.2)	7.41 ± 0.63		5.39 ± 0.71	
Nurse	22 (6.4)	6.50 ± 1.21		4.86 ± 0.87	
Pharmacist	16 (4.6)	6.56 ± 1.47		4.44 ± 1.17	
MLS	20 (5.8)	6.90 ± 1.13		5.70 ± 0.50	
Others	52 (15.0)	6.96 ± 0.96		4.81 ± 0.71	
**Geopolitical Zone**					
North Central	104 (30.1)	7.25 ± 0.43	0.479	5.19 ± 0.33	0.053
Northeast	113 (32.7)	6.95 ± 0.65		4.96 ± 0.58	
Northwest	30 (8.7)	7.03 ± 0.83		5.50 ± 0.67	
Southeast	27 (7.8)	7.59 ± 0.65		5.63 ± 0.30	
South south	37 (10.7)	7.19 ± 0.90		5.65 ± 0.28	
Southwest	35 (10.1)	7.06 ± 0.50		5.06 ± 0.49	
**Marital status**					
Married	242 (69.9)	6.47 ± 2.25	0.88	5.29 ± 0.39	0.824
Unmarried	104 (30.1)	6.18 ± 2.26		5.35 ± 0.41	
**Religion**					
Christianity	244 (70.5)	7.21 ± 0.46^a^∗	**0.019**	5.38 ± 0.40	0.359
Islam	96 (27.7)	7.00 ± 0.62		5.10 ± 0.68	
No religion	6 (1.7)	6.00 ± 1.23^b^∗		5.67 ± 0.82	
**Overall Score (%)**		**7.1 (88.8)**		**5.30 (88.5)**	

Mean score with the different letters in the same column were significantly different (*P* > 0.05), ∗ = p > 0.05, ∗∗p > 0.01and ∗∗∗p > 0.001.

### 3.3 Attitudes of healthcare workers towards COVID-19

The attitude score on a scale of 0–6 varies significantly across age groups (p = 0.014) and years of experience in healthcare service (p = 0.001). Healthcare workers within the age group 50 and above had an attitude score of 4.57 ± 0.60, which was lower than HCWs in other age categories combined. Also, those who had 31 and above years of experience in healthcare services had a lower attitude score (3.71 ± 1.04) compared with those with lower years of experience (Table 3). The rate of positive attitude towards COVID-19 ranges from 271 (78.3%) to 326 (94.2%).

Table 4 showed Healthcare workers' demographic characteristics and attitudes towards COVID-19. Although there was a statistically significant difference among the age group (χ^2^ = 18.360, p = 0.005) and health professionals (χ^2^ = 21.501; p = 0.001), a vast majority (92.2%) believed that COVID-19 was not a biological weapon against people in African. The age group 26–30 and work experience for 31 and above believed that SARS-COV-2 was invented to reduce the African population was 22.2% and 28.6%, respectively. Also, among health professionals, Nurses (13.6%) and pharmacists (37.5%) agreed that COVID-19 was a biological weapon against Africans. The majority (78.3%) of participants agreed that faith healing is not the only cure for COVID-19. When asked concerning faith healing as the only cure for COVID-19, it was observed that there was significant difference across years of experience (χ^2^ = 33.311; p = 0.000), health professionals (χ^2^ = 37.120 (0.000) and geopolitical zones (χ^2^ = 13.563; p = 0.019). The attitude of HCWs towards congregating in large crowd differs significantly across age group (χ^2^ = 12.925; p = 0.005), years of experience (χ^2^ = 19.447; p = 0.003) and health professionals (χ^2^ = 18.018; p = 0.006). The majority (92.5%) of the participants in this study have confidence that medical scientists will defeat the COVID-19 pandemic. The response toward overcoming COVID-19 pandemic differs significantly across age group (χ^2^ = 35.749; p = 0.000), years of service (χ^2^ = 14.313; p 0.003), health professionals (χ^2^ = 24.617; p = 0.000) and geopolitical zones (χ^2^ = 11.201; p = 0.048).

**Table 4:** Attitudes of Healthcare workers towards COVID-19 in Nigeria.

Demographic Characteristics	COVID-19 was invented to reduce the human population in Africa			Faith/Spiritual healing is an only cure for COVID-19			Congregation/large crowd at a place of worship			∗Confidence that medical scientists overcome SAR-CoV-2 virus		
	False (%)	True (%)	χ^2^ (P- value)	True (%)	False (%)	χ^2^ (P - value)	True (%)	False (%)	χ^2^ (P- value)	True (%)	False (%)	χ^2^ (P - value)
**Gender**												
Male	228 (91.6)	21 (8.4)	0.490 (0.484)	52 (20.9)	197 (79.1)	0.329 (0.566)	15 (6.0)	234 (94.0)	0.097 (0.756)	233 (93.6)	16 (6.4)	1.515 (0.218)
Female	91 (93.8)	6 (6.2)		23 (23.7)	74 (76.3)		5 (5.2)	92 (94.8)		87 (89.7)	10 (10.3)	
**Age group (years)**												
20–29	34 (94.4)	2 (5.6)	4.135 (0.247)	5 (13.9)	31 (86.1)	2.518 (0.472)	4 (11.1)	32 (88.9)	**12.925 (0.005)**	34 (94.4)	2 (5.6)	**35.749 (0.000**)
30–39	199 (93.0)	15 (7.0)		45 (21.0)	169 (79.0)		6 (2.8)	208 (97.2)		201 (93.9)	13 (6.1)	
40–49	69 (92.0)	6 (8.0)		19 (25.3)	56 (74.9)		6 (8.0)	69 (92.0)		70 (93.3)	5 (6.7)	
50–59	17 (81.0)	4 (19.0)		6 (28.6)	15 (71.4)		4 (19.0)	17 (81.0)		15 (71.4)	6 (28.6)	
**Experience (year)**												
0–5	82 (93.2)	6 (6.8)	**18.360 (0.005)**	29 (33.0)	59 (67.00	**33.311 (0.000)**	7 (8.0)	81 (92.0)	**19.447 (0.003)**	77 (87.5)	11 (12.5)	**14.313 (0.003)**
6–10	139 (95.9)	6 (4.1)		15 (10.3)	130 (89.7)		4 (2.8)	141 (97.2)		141 (97.2)	4 (2.8)	
11–15	52 (89.7)	6 (10.3)		17 (29.3)	41 (70.7)		3 (5.2)	55 (94.8)		56 (96.6)	2 (3/4)	
16–20	13 (81.2)	3 (18.8)		4 (25.0)	12 (75.0)		2 (12.5)	14 (87.5)		15 (93.8)	1 (6.2)	
21–25	16 (100)	0 (0.0)		2 (12.5)	14 (87.5)		0 (0.0)	16 (100)		14 (87.5)	2 (12.5)	
26–30	7 (77.8)	2 (22.2)		0 (0.0)	9 (100)		0 (0.0)	9 (100)		9 (100)	0 (0.0)	
31+	10 (71.4)	4 (28.6)		8 (57.1)	6 (42.9)		4 (28.6)	10 (71.4)		8 (57.1)	6 (42.9)	
**Health Profession**												
Medical doctor	64 (95.5)	3 (4.5)	**21.501 (0.001)**	12 (17.9)	55 (82.1)	**37.12. (0.000)**	1 (1.5)	66 (98.5)	**18.018 (0.006)**	**64 (95.5)**	**3 (4.5)**	**24.617 (0.000)**
Veterinary doctor	112 (93.3)	8 (6.7)		15 (12.5)	105 (97.5)		6 (5.0)	114 (95.0)		113 (94.2)	7 (5.8)	
Public Health	46 (93.9)	3 (6.1)		5 (10.20	44 (89.8)		1 (2.0)	48 (98.0)		49 (100)	0 (0.0)	
Nurse	19 (86.4)	3 (13.6)		9 (40.9)	13 (59.1)		2 (9.1)	15 (93.8)		17 (77.3)	5 (22.7)	
Pharmacist	10 (62.5)	6 (37.5)		9 (56.2)	7 (43.8)		1 (6.2)	15 (93.8)		15 (93.8)	1 (6.2)	
MLS	20 (100.0)	0 (0.0)		4 (20.0)	16 (80.0)		0 (0.0)	9 (100)		20 (100)	0 (0.0)	
Others	48 (92.3)	4 (7.7)		21 (40.1)	31 (59.9)		9 (17.3)	43 (82.7)		42 (80.8)	10 (19.2)	
**Geopolitical Zone**												
North Central	99 (95.2)	5 (4.8)	7.137 (0.211)	19 (18.3)	85 (81.7)	**13.563 (0.019)**	5 (4.8)	99 (95.2)	3.735 (0.588)	**101 (97.1)**	**3 (2.9)**	**11.201 (0.048)**
Northeast	101 (89.4)	12 (10.6)		34 (30.1)	79 (69.9)		9 (8.0)	104 (92.0)		98 (86.7)	15 (13.3)	
Northwest	30 (100)	0 (0.0)		2 (6.7)	28 (93.3)		0 (0.0)	30 (100)		28 (93.3)	2 (6.7)	
Southeast	25 (92.6)	2 (7.4)		4 (14.8)	23 (85.2)		1 (3.7)	26 (96.3)		26 (96.3)	1 (3.7)	
Southsouth	34 (91.9)	3 (8.1)		5 (13.5)	32 (86.5)		2 (5.4)	35 (94.6)		36 (97.3)	1 (2.7)	
Southwest	30 (85.7)	5 (14.3)		11 (31.4)	24 (68.6)		3 (8.6)	32 (91.4)		31 (88.6)	4 (11.4)	
**Marital status**												
Married	223 (92.1)	19 (7.9)	0.003 (0.960)	51 (21.1)	191 (78.9)	0.172 (0.679)	17 (7.0)	225 (93.0)	2.289 (0.130)	225 (93.0)	17 (7.0)	0.278 (0.598)
Unmarried	96 (94.3)	8 (7.7)		24 (23.1)	80 (76.9)		3 (2.9)	101 (97.1)		95 (91.3)	9 (8.7)	
**Religion**												
Christianity	227 (93.0)	17 (7.0)	1.656 (0.437)	58 (23.8)	186 (76.2)	3.181 (0.204)	15 (6.1)	229 (93.9)	0.496 (0.784)	**227 (93.0)**	**17 (7.0)**	5.867 (0.053)
Islam	86 (89.6)	10 (10.4)		17 (17.7)	79 (82.3)		5 (5.2)	91 (94.8)		89 (92.7)	7 (7.3)	
No Religion	6 (100)	0 (0.0)		0 (0.0)	6 (100)		0 (0.0)	6 (100)		4 (66.7)	2 (33.3)	
**Total**	**319 (92.2)**	**27 (7.8)**		**271 (78.3)**	**75 (21.7)**		**326 (94.2)**	**20 (5.8)**		**320 (92.5)**	**26 (7.5)**	

∗ Adapted from Zhong et al., 2020; + Public Health include Community health and Veterinary Public health officer.

### 3.4 Practices of healthcare workers towards COVID-19

Overall, most of the participants agreed that personal hygiene measures could reduce the risk of SARS-COV-2 infections. Washing hands after handling sick persons/animals (96.0%), wearing personal protective equipment (PPE) (91.6%), washing of hands after handling pets/companion animals (96.0%), and proper covering of mouth when coughing and sneezing (90.5%). Generally, the practice of wearing a face mask when leaving home (59.2%) among HCW in this study was poor (Table 2).

There were significant (χ^2^ = 21.501, P = 0.001) differences among healthcare professionals with regards to the washing of hands after handling infected persons or animals. One hundred per cent of veterinary doctors and pharmacists said that they wash their hands after handling sick animals or persons. However, Nurses (18.2%) and Medical Laboratory Scientist (10.0%) do not wash hands after handling ill persons or animals. There were significant differences (χ^2^ = 8.753, P = 0.000) among healthcare professionals on the issue of wearing PPE when handling specimens from persons with COVID-19. Medical doctors (20.9%) and Nurses (22.7%) do wear PPE when handling specimens from persons with signs of COVID-19. Furthermore, we also observed significant differences (χ2 = 14.275, P = 0.027) among the different health professionals concerning the use of a face mask when leaving home. Medical doctors (53.2%) Veterinary doctors (55.8%) and others (42.3%) had poor practice on the use of face masks when leaving home (Table 5).

**Table 5:** Practices of Healthcare workers towards COVID-19 in Nigeria.

Variables	Do you wash your hands after handling your pet or companion animal?			Do you wear protective clothing when handling a specimen from a person with signs of dry cough, sneezing, and fever?			Do you wear a mask when leaving home?		
Demographic Characteristics	Yes (%)	No (%)	χ^2^ (P value)	Yes (%)	No (%)	χ^2^ (P value)	Yes (%)	No (%)	χ^2^ (P value)
Gender									
Male	239 (96.0)	10 (4.0)	0.002 (0.964)	233 (93.6)	16 (6.4)	**4.424 (0.035)**	141 (56.6)	108 (43.4)	2.529 (0.116)
Female	93 (95.9)	4 (4.1)		84 (88.6)	13 (13.4)		64 (66.0)	33 (34.0)	
Age group (years)									
20–29	35 (97.2)	1 (2.8)	1.369 (0.713)	31 (86.1)	5 (13.9)	6.881 (0.076)	22 (61.1)	14 (38.9)	0.120 (0.989)
30–39	205 (95.8)	9 (4.2)		193 (90.2)	21 (9.8)		126 (61.8)	88 (41.1)	
40–49	71 (94.7)	4 (5.3)		74 (98.7)	1 (1.3)		45 (60.0)	30 (40.0)	
50–59	21 (100)	0 (0.0)		19 (90.5)	2 (9.5)		12 (57.1)	9 (42.9)	
Experience (year)									
0–5	86 (97.7)	2 (2.3)	8.538 (0.201)	79 (89.8)	9 (10.2)	5.811 (0.445)	43 (48.9)	45 (51.1)	8.412 (0.209)
6–10	140 (96.6)	5 (3.4)		131 (90.3)	14 (9.7)		94 (64.8)	51 (35.2)	
11–15	56 (96.6)	2 (2.4)		56 (96.6)	2 (3.4)		36 (62.1)	22 (37.9)	
16–20	14 (87.5)	2 (12.5)		16 (100)	0 (0.0)		7 (43.8)	9 (56.2)	
21–25	14 (87.5)	2 (12.5)		14 (87.5)	2 (12.5)		11 (68.8)	5 (31.2)	
26–30	8 (88.9)	1 (11.1)		9 (100)	0 (0.0)		6 (66.7)	3 (33.3)	
31+	14 (100)	0 (0.0)		12 (85.7)	2 (14.3)		8 (57.1)	6 (42.9)	
Health Profession									
Medical doctor	62 (92.5)	5 (7.5)	**21.501 (0.001)**	53 (79.1)	14 (20.9)	**28.753 (0.000)**	39 (58.2)	28 (41.8)	**14.275 (0.027)**
Veterinary doctor	120 (100)	0 (0.0)		116 (96.7)	4 (3.3)		67 (55.8)	53 (44.2)	
Public Health	47 (95.9)	2 (4.1)		48 (98.0)	1 (2.0)		36 (73.5)	13 (26.5)	
Nurse	18 (81.8)	4 (18.2)		17 (77.3)	5 (22.7)		15 (68.2)	7 (31.8)	
Pharmacist	16 (100)	0 (0.0)		14 (87.5)	2 (12.5)		11 (68.8)	5 (31.2)	
MLS	18 (90.0)	2 (10.0)		20 (100)	0 (0.0)		15 (75.0)	5 (25.0)	
Others	51 (98.1)	1 (1.9)		49 (94.2)	3 (5.8)		22 (42.3)	30 (57.7)	
Geopolitical Zone									
North Central	101 (97.1)	3 (2.9)	9.325 (0.097)	94 (90.4)	10 (9.6)	8.711 (0.121)	51 (49.0)	53 (51.0)	**21.158 (0.001)**
Northeast	109 (96.5)	4 (3.5)		108 (95.6)	5 (4.4)		66 (58.4)	47 (41.6)	
Northwest	30 (100)	0 (0.0)		29 (96.7)	1 (3.3)		20 (66.7)	10 (33.3)	
Southeast	27 (100)	0 (0.0)		22 (81.5)	5 (18.5)		26 (96.3)	1 (3.7)	
Southsouth	34 (91.9)	3 (8.1)		34 (91.9)	3 (8.1)		20 (54.1)	17 (45.9)	
Southwest	31 (88.6)	4 (11.4)		30 (85.7)	5 (14.3)		22 (62.9)	13 (37.1)	
Marital status									
Married	234 (96.7)	8 (3.3)	1.137 (0.286)	226 (93.4)	16 (6.6)	3.284 (0.070)	145 (59.9)	97 (40.1)	0.149 (0.699)
Unmarried	98 (94.2)	6 (5.8)		91 (87.5)	13 (12.5)		60 (57.7)	44 (42.3)	
Religion									
Christianity	235 (96.3)	9 (3.7)	**13.546 (0.001)**	225 (92.2)	19 (7.8)	4.977 (0.083)	137 (56.1)	107 (43.9)	**6.230 (0.044)**
Islam	93 (96.9)	3 (3.1)		88 (91.7)	8 (8.3)		62 (64.6)	34 (35.4)	
No Religion	4 (66.7)	2 (33.3)		4 (66.7)	2 (33.3)		6 (100)	0 (0.0)	
**Total**	**332 (96.0)**	**14 (4.0)**		**317 (91.6)**	**26 (8.4)**		**205 (59.2)**	**141 (40.8)**	

Table 6 shows the relationships between knowledge, attitudes, and practice. There was significantly positive correlation (r = 0.584; p = 0.046) between knowledge and attitude. Further details are presented in the supplementary data (Suppl.1).

**Table 6:** Correlation between scores of knowledge, attitude, and practice.

Variables	Correlation coefficient	P-value
Knowledge–Attitudes	0.584	0.046
Knowledge–Practice	0.090	0.804
Attitude–Practice	0.338	0.339

Correlation is significant at the 0.05.

## 4 Discussion

To the best of our knowledge, this study is among the first studies in Nigeria that thoroughly assessed the knowledge, attitude, and practice of Healthcare workers (HCWs) towards COVID-19. In this study, over 70% of the respondents were male, more than 30% were from north-central and north-eastern Nigeria, respectively. Veterinary doctors (34.7%) and medical doctors (19.4%) had the highest participants in the study.

Our findings showed that most HCWs in Nigeria had a good understanding of COVID-19. The outcome is not unrelated to the current national response activities by the Nigerian Center for Disease Control and Prevention/Presidential Task Force on COVID-19 (NCDC/PTF). The mean knowledge score by the respondents was 7.1, representing 88.8% (7.1/8). The mean knowledge score was significantly (p > 0.05) lowest among respondents who do not have religion. The reason for the difference in mean knowledge score among religious and non-religious adherents was beyond the scope of the study scope and off-course for a reason not clearly understood. However, the mean knowledge score in this study was not statistically different among the different age groups and categories of years of work experience. However, a study in Egypt reported that the age group 50 and above had a lower mean knowledge score of COVID-19 [17]. A previous study in Pakistan reported significant (p > 0.05) difference among age groups for the COVID-19 mean knowledge score [20]. A study among internet users in Bangladesh reported a high mean knowledge score among age groups 30 years and above compared to other age categories [21].

The knowledge about COVID-19 was gained by respondents mostly through television and social media. The improvement in technology and the ease of accessing information online and television may explain the high level of knowledge of HCWs in this study that relied on these means of communication to obtain information about COVID-19. This result was consistent with previous studies in Nigeria [22, 23]. The wide media coverage of COVID-19 by WHO, Federal and state government in Nigeria, and strict preventive and control measures imposed by the government and its agencies (NCDC) may be responsible for the high mean knowledge score by respondents in this study. Also, the practice of posting and reposting of professional information in the social media group page such as WhatsApp, Facebook, and Twitter accounts may be a second reason. Currently, there is no treatment against COVID-19 globally; HCWs play a vital role in the management, control, and prevention of the spread of the disease. Good knowledge of HCWs on the transmission and preventive measures against SAR-CoV-2 can help to improve their skills to limit occupational risk and further spread to the community [24]. However, precautions are necessary when using social media as sources of medical knowledge because of information overload [25], lack of peer review, and misleading information. A previous study reported the possibility that there exist dual pandemic, COVID-19 pandemic, and a pandemic of infodemic [20], which may seriously jeopardize response efforts to contain the spread of the virus. The result obtained in this study were similar to the previous report in China [18]; Italy [26]; Egypt [17]; Pakistan [20], and Nigeria [22, 23], in which social media and television served as the major sources of COVID-19 information.

The attitudes of HCWs influence their practice of infection prevention, and control measures against COVID-19. In this study, we assessed the attitude of HCWs about their acceptance of beliefs and misconceptions. Such as African are immune to COVID-19 because of their genetic makeup, SARS-CoV-2 was a biological weapon to reduce the population of Africa, COVID-19 is a punishment from God because people have sinned. Faith healing or prayer is the only cure for COVID-19. The acceptance of these negative attitudes in the community by HCWs may jeopardize the effort to contain the spread of the disease. Also, the community looks up to HCWs as role models in respect of practices and attitudes toward health.

Our finding revealed that 88.5% of the study participants demonstrated a positive attitude towards COVID-19. There was significant (p = 0.014) differences among age groups and mean attitude scores. Age group 50–59 years had the lowest mean attitude score compare to the age group 20–39 years. Our findings revealed that the mean attitude score differs significantly (p > 0.05) concerning years of experience. Those who had 31 and above years of experience had the lowest mean attitude score. The differences in mean attitude score may be because young HCWs with few years of experience are more likely to seek new knowledge than the older HCWs with long years of experience. Our findings differ with the previous study in Pakistan in which the mean attitude score was found not to differ significantly (p < 0.05) with age or years of experience in healthcare services [20]. Interestingly, our study revealed a significant correlation between knowledge score and attitude. This finding is consistent with the report by Saqlain et al. [20].

Despite the effort of the WHO and relevant government agencies on the origin of SARS-Cov-2, 22.2% and 28.6% of HCWs of 26–30 and 31 and above years of working experience believed that SARS-CoV-2 was a biological weapon against Africans. Also, 37% of pharmacists said that COVID-19 was a biological weapon. Generally, 27 (7.8%) of the respondents agreed that SARS-CoV-2 was a biological weapon. This finding reflects the damaging effects of bad rumours and misconceptions. False information was circulating on social media and the internet in Nigeria, claiming that COVID-19 was a biological weapon to reduce the African population. In another rumour, some researchers claimed that SARS-CoV-2 was created in laboratories (Praghan et al., 2020). An article titled "The Proximal Origin of SARS-CoV-2" [2] dismissed the misconception that SARS-CoV-2 was a deliberate weapon. In a similar study in Egypt, Abdelhafiz et al. [17] reported a higher percentage (26.8%) of respondents who thought that SARS-CoV-2 was a biological weapon created in a laboratory. The study further observed that misinformation aided by social media and the internet were the likely reason for the high rate.

About 21.7% of the respondents thought that faith healing or prayers are the only cure for COVID-19. This number, although small, reflects the religious nature of the study participants. Nigerians are known to be so religious that they attribute religious motifs to almost everything that they do. Furthermore, their religiousness exemplified the way they view and respond to issues and realities in the world [27, 28]. Concerning attitude towards congregation in a large crowd at a place of worship, 94.2% of the respondents thought that it is wrong for people to gather in large crowds at places of worship. This result is a relevant finding because of the nature of transmission of COVID-19 [1,29,30]. The large congregation makes it easier for an infectious case to spread SARS-CoV-2 in crowded environments such as places of worship, markets, bus stations, train stations, and schools, [18, 29]. A similar finding was reported among the general public in China, where respondents agreed that avoiding crowded places is an important preventive measure against the spread of SARS-CoV-2 [18]. The study demonstrated the willingness of HCWs to adapt to new changes and lifestyles in the face of the global pandemic.

A large majority of the HCWs held positive attitudes towards winning the war against COVID-19. About 9 out of 10 respondents were confident that medical scientists in Nigeria would overcome COVID-19. The explanation for the high level of confidence by the HCWs may be because of the success of Nigerian HCWs in tackling previous outbreaks like Ebola virus in 2014, the Avian influenza virus in 2007, and Rinderpest in the 80s [31, 32, 33, 34].

Although our findings did not report significant (p < 0.05) correlation between knowledge and practice, the results showed that HCWs in this study demonstrated good practice towards COVID-19. When asked regarding measures that might help to contain the spread of SARS-CoV-2, there was strong agreement among HCWs that washing of hands after handling pets or companion animals (96.0%) and wearing of personal protective equipment when handling specimen from suspected COVID-19 patient (91.6%) might help to prevent transmission. Although, as at the time of writing this article, the role of animals in the transmission of SAR-CoV-2 is yet unknown, people still engaged in a practice that can prevent transmission from their pets and companion animals. Concerning the washing of hands, 18.2% of nurses and 10.0% of medical laboratory scientists do not wash their hands after handling their pets or companion animals. The proportion of female 13 (13.4%) who do not wear PPE when handling specimens from suspected COVID-19 patients was significantly (χ^2^ = 4.420; p = 0.035) higher than male 16 (6.4%). The difference observed in this study may be because of the difference in the availability and accessibility of PPE. In the face of scarcity, women are often at a disadvantage than men [35, 36]. Males in Nigeria are considered to have control over resources than females, unlike some Asian countries where women have more wealth than men [18].

In a similar study in China, females observed preventive measures than males against COVID-19 [18]. In another study in the United States, those who are living below the poverty level have a low perception of personal risk and limited ability to prevent infection [37]. Our finding showed that a large proportion of medical doctors (20.9%) and nurses (22.7%) do not use PPE when handling specimens from suspected COVID-19 patients. The reason for this practice among medical doctors and nurses is unclear. However, previous studies reported that allied healthcare workers have a higher knowledge of preventive practices against infectious diseases than medical doctors [20, 25].

When asked concerning the use of a face mask when leaving home, only 3 out of 5 respondents said that they use a face mask when leaving home. Our results agreed with a previous study in Malaysia, where the proportion of those who use a face mask when leaving home was above 50% [38]. The reason for this observation maybe because of the scarcity and cost of face mask during the first week of the COVID-19 outbreak in Nigeria, and maybe a reflection of the global scarcity of face mask brought about by high demand and low production as a result of global lockdown [38, 39]. The CDC recommended the use of cloth face mask for the public [40, 41]. Another measure to manage the scarcity of face mask for use by health care workers by WHO was the recommendation that only COVID-19 patients with respiratory symptoms or COVID-19 caregivers should use face mask [39]. In Nigeria, the presidential task force (PTF) on COVID-19 recommended the use of face masks by all irrespective of status. However, the PTF did not specify the type of face mask to be used by the general public. Though the standard recommended use of face mask is based on the risk assessment of healthcare workers.

## 5 Conclusion

Our findings revealed that HCWs in Nigeria have good knowledge about COVID-19, and possess a positive attitude and good preventive practice to contain the transmission of SARS-CoV-2. The familiar sources of information on COVID-19 knowledge were social media and television. However, the practice of wearing a face mask when leaving home was poor among HCWs in this study. Also, the attitude among older HCWs and those with 31 and above years of experience was low. The results showed that there exists a positive correlation between knowledge and attitude and knowledge and practice. We recommended that public health education be targeted towards the categories of HCWs with high-risk practices and attitudes to achieve the necessary control measure been instituted by the government (risk assessment).

## 6 Limitation

Accessibility and availability of internet services in Nigeria is limited. Therefore, only those who can have access to the available internet were able to participate in the survey. The survey was only available on the contacts of the authors and those who use WhatsApp, Twitter, Facebook, and the internet. These represent a significant challenge to this study.

## Declarations

### Author contribution statement

F. Ejeh: Conceived and designed the experiments; Performed the experiments; Analyzed and interpreted the data; Contributed reagents, materials, analysis tools or data; Wrote the paper.

A.S. Saidu and S. Owoicho: Performed the experiments; Analyzed and interpreted the data; Contributed reagents, materials, analysis tools or data; Wrote the paper.

N.A. Maurice and K. Okon: Performed the experiments; Analyzed and interpreted the data; Wrote the paper.

S. Jauro and L. Madukaji: Performed the experiments; Wrote the paper.

### Funding statement

This research did not receive any specific grant from funding agencies in the public, commercial, or not-for-profit sectors.

### Declaration of interests statement

The authors declare no conflict of interest.

### Additional information

No additional information is available for this paper.

## Appendix A Supplementary data

COVID-19_KAPs_NIGERIA_SUPPLIMENTARY DATA COVID-19_KAPs_NIGERIA_SUPPLIMENTARY DATA
